# Continuous Microfluidic Production of Citrem-Phosphatidylcholine Nano-Self-Assemblies for Thymoquinone Delivery

**DOI:** 10.3390/nano11061510

**Published:** 2021-06-07

**Authors:** Esra Ilhan-Ayisigi, Aghiad Ghazal, Barbara Sartori, Maria Dimaki, Winnie Edith Svendsen, Ozlem Yesil-Celiktas, Anan Yaghmur

**Affiliations:** 1 Department of Bioengineering, Faculty of Engineering, Ege University, 35100 Bornova-Izmir, Turkey; esrailhan01@gmail.com (E.I.-A.); ozlem.yesil.celiktas@ege.edu.tr (O.Y.-C.); 2 Genetic and Bioengineering Department, Faculty of Engineering and Architecture, Kirsehir Ahi Evran University, 40100 Kirsehir, Turkey; 3 Department of Pharmacy, Faculty of Health and Medical Sciences, University of Copenhagen, Universitetsparken 2, DK-2100 Copenhagen, Denmark; aghiadghazal@gmail.com; 4 Global Research Technologies, Novo Nordisk, Novo Nordisk Park, 2760 Måløv, Denmark; 5 Institute of Inorganic Chemistry, Graz University of Technology, Stremayrgasse 9/4, 8010 Graz, Austria; barbara.sartori@tugraz.at; 6 DTU Bioengineering—Department of Biotechnology and Biomedicine, Technical University of Denmark, Søltofts Plads Bldg. 221, 2800 Kongens Lyngby, Denmark; madi@dtu.dk (M.D.); wisv@dtu.dk (W.E.S.)

**Keywords:** thymoquinone, inverse bicontinuous cubic *Pn3m* phase, microfluidics, nanoparticle tracking analysis, synchrotron small-angle scattering

## Abstract

Lamellar and non-lamellar liquid crystalline nanodispersions, including liposomes, cubosomes, and hexosomes are attractive platforms for drug delivery, bio-imaging, and related pharmaceutical applications. As compared to liposomes, there is a modest number of reports on the continuous production of cubosomes and hexosomes. Using a binary lipid mixture of citrem and soy phosphatidylcholine (SPC), we describe the continuous production of nanocarriers for delivering thymoquinone (TQ, a substance with various therapeutic potentials) by employing a commercial microfluidic hydrodynamic flow-focusing chip. In this study, nanoparticle tracking analysis (NTA) and synchrotron small-angle X-ray scattering (SAXS) were employed to characterize TQ-free and TQ-loaded citrem/SPC nanodispersions. Microfluidic synthesis led to formation of TQ-free and TQ-loaded nanoparticles with mean sizes around 115 and 124 nm, and NTA findings indicated comparable nanoparticle size distributions in these nanodispersions. Despite the attractiveness of the microfluidic chip for continuous production of citrem/SPC nano-self-assemblies, it was not efficient as comparable mean nanoparticle sizes were obtained on employing a batch (discontinuous) method based on low-energy emulsification method. SAXS results indicated the formation of a biphasic feature of swollen lamellar (L_α_) phase in coexistence with an inverse bicontinuous cubic *Pn3m* phase in all continuously produced TQ-free and TQ-loaded nanodispersions. Further, a set of SAXS experiments were conducted on samples prepared using the batch method for gaining further insight into the effects of ethanol and TQ concentration on the structural features of citrem/SPC nano-self-assemblies. We discuss these effects and comment on the need to introduce efficient microfluidic platforms for producing nanocarriers for delivering TQ and other therapeutic agents.

## 1. Introduction

Cubosomes and hexosomes are analogous to liposomes, which are widely used as drug nanocarriers since their discovery a half-century ago [1], but have different structural properties as they envelope three- (3D) and two-dimensional (2D) self-assembled interiors, respectively [2,3,4,5,6,7,8,9,10,11,12]. In the development of nanocarriers for drug delivery, the attractiveness of these lipid nanoparticles and related nano-self-assemblies including micellar cubosomes and emulsified microemulsions (EMEs) is linked to their capability of loading drugs and imaging agents with different physiochemical properties [3,5,13,14,15,16,17,18,19,20,21]. The localization of these agents in the nanoparticles’ interiors is governed by a set of complex interactions of the loaded guest molecules with the main lipid constituents and water [3,6,13,15,22,23,24,25,26]. Accordingly, water-soluble and poorly water-soluble drugs or imaging probes will tend to be accommodated into the hydrophilic and hydrophobic domains of the self-assembled interiors, respectively, whereas amphiphilic drugs tend to be localized at the lipid-water interfaces [3,7,13,14,27,28,29].

In a recent study [30], a structurally tunable and immune safe library of colloidally stable lamellar and non-lamellar liquid crystalline (LC) nanoparticles based on binary lipid mixtures of soy phosphatidylcholine (SPC) and citrem was introduced. The latter is a well-known FDA-approved anionic food-grade emulsifier that is typically used to stabilize emulsions, and lamellar and non-lamellar liquid crystalline nanoparticles [30,31]. Citrem/SPC nanoparticles are hemocompatible as indicated by a lack of complement activation and subsequent hemolytic effects [24,25], and therefore are attractive for use in the development of drug nanocarriers. In excess water, incorporation of citrem into SPC-water interfacial area is associated in a concentration-dependent manner with lamellar-nonlamellar structural transitions, leading to the production of vesicles, cubosomes, and hexosomes by using either low-energy (vortexing) or high-energy emulsification batch methods [28,30,32,33]. However, there is still no report on the continuous production of these nano-self-assemblies by using microfluidic platforms. As compared to most investigated cubosomes and hexosomes based on unsaturated monoglycerides [2,5,6,7,34], citrem is attractive for use in the production of immune safer non-lamellar liquid crystalline nanoparticles based on SPC, which is a well-known phospholipid with propensity to form a lamellar (L_α_) liquid crystalline phase in excess water [30].

Unlike liposomes [35,36,37,38,39,40] and other lipid nanoparticles [41,42,43], the continuous microfluidic synthesis of cubosomes, hexosomes, and other related nano-self-assemblies is rarely investigated [44,45]. Among different microfluidic mixers, hydrodynamic flow focusing (HFF) microfluidic platforms are the most employed for the continuous production of these nanoparticles [35,39,46,47,48,49]. They are attractive for use owing to their capability of controlling the mean nanoparticle size and size distribution by creating a well-defined and predictable interfacial region between the injected fluids, and hydrodynamically focusing of the centre and side streams to submicrometer-sized dimensional scales for rapid mixing and patterning [35,38,39,40,45].

In the following, we report on continuous production of citrem/SPC nano-self-assemblies by using a commercial hydrodynamic flow focusing (HFF) microfluidic chip, and explore the possible use of these nano-self-assemblies as nanocarriers for delivering thymoquinone (TQ), which is the main therapeutic compound (30–48%) of the essential volatile oil of *Nigella sativa* and has wide therapeutic potentials [16,50,51]. In literature, various nanoparticles including liposomes [52], micellar cubosomes [16], niosomes [53], and albumin [54] nanoparticles were suggested for delivering TQ and overcoming different reported limitations (including poor bioavailability and membrane penetration ability) [16,54]. In the present study, we report also on the effects of ethanol concentration and lipid composition on the structural features and size characteristics of the continuously produced citrem/SPC nanoparticles (samples A1–A8, Table 1) by using synchrotron small-angle X-ray scattering (SAXS) and nanoparticle tracking analysis (NTA), respectively. For evaluating the efficiency of the microfluidic platform at same ethanol concentrations and lipid compositions, the mean nanoparticle sizes and size distributions of two selected continuously produced citrem/SPC nanoparticles were compared with those generated by using a batch emulsification method. The latter method is based on vortexing the binary citrem/SPC mixtures in excess water for 5 min (samples A9 and A10, Table 1).

## 2. Experimental Section

### 2.1. Materials

Soy phosphatidylcholine (SPC, 97.6% purity) was purchased from Lipoid GMBH (Ludwigshafen, Germany). Grinsted^®^ citrem LR10, a combination of citric acid esters of mono- and di-glycerides made from sunflower oil, was received as a gift from Danisco A/S (Copenhagen, Denmark). It is synthesized from sunflower oil and contains 64% of mono- and di-glycerides, 36% of glycerol citrate fatty acid esters in addition to traces of triacylglycerides and free fatty acids [55]. Thymoquinone (≥98% purity) and phosphate buffered saline (PBS) tablets at pH 7.4 were purchased from Sigma Aldrich (Poole, United Kingdom). Ethanol (EtOH) with a purity of 96% was purchased from Merck Millipore (Darmstadt, Germany). For preparing the 0.01 M PBS buffer at pH 7.4, Milli-Q water was used (Millipore Direct-Q3 UV system, Billerica, MA, USA). All materials were used without further purification.

### 2.2. Continuous Production of Citrem/SPC Nano-Self-Assemblies

**Figure 1:**
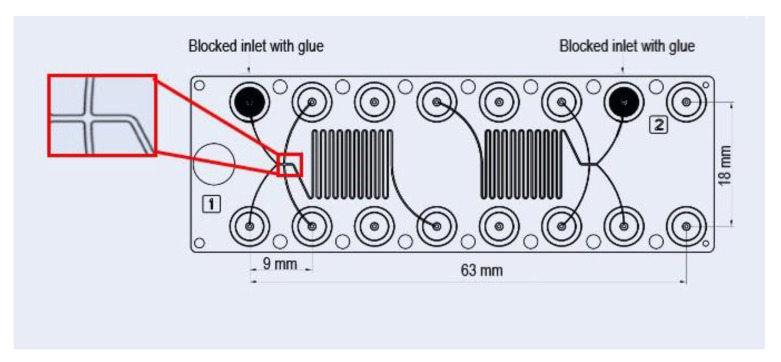
A schematic representation of the used commercial microfluidic hydrodynamic flow focusing polycarbonate chip. The dimensions of the microchannels are 100 µm depth, 100 µm core inlet channel width, 200 µm lateral inlet channel width, 200 µm mixer channel width, and 200 µm output channel width.

The continuous microfluidic synthesis of TQ-free and TQ-loaded nanodispersions was achieved by using the commercial Fluidic 186 HFF chips purchased from *microfluidic ChipShop* (Jena, Germany). In these diffusion micromixers, two independent HFF polycarbonate (PC) chips are placed on the same microfluidic platform (Figure 1), and each chip has two lateral and two core flow inlets. However, one of these core inlets was blocked with glue for producing the nanoparticles by using a three-inlet hydrodynamic flow-focusing microfluidic chip. The continuous production of TQ-free nanoparticles was achieved by injecting the two sheath (side) fluids (PBS streams) through the lateral inlets and hydrodynamically focusing and confining the ethanol solution of citrem/SPC, which was injected through the single core inlet of the HFF chip. In this method, the already prepared stock ethanol solution of 40 wt% lipid (a binary citrem/SPC mixture prepared at citrem/SPC weight ratio of 2:3) and 60 wt% ethanol was loaded into 1 mL syringe (B. Braun Injects-F Solo syringe, Melsungen, Germany), whereas PBS was loaded into two 5 mL BD conventional syringes with a BD Luer-Lok™ tip (BD, Franklin Lakes, NJ, USA). The syringes were connected to the microfluidic device by tubes and blunt needles (Terumo mixing needle without bevel, 18 G × 1.5”, 1.2 × 40 mm), and mounted on the pump (KR Analytical Ltd., Sandbach, Cheshire, UK). In this set-up, the employed total flow rate (TFR) is the sum of the flow rates of the central and side streams; whereas FRR (flow rate ratio) is the ratio of the flow rates of the side streams to that of the central stream. In this procedure, the continuous production of the nanoparticles was achieved at two different TFRs (50 and 100 µL/min) and a constant FRR of 20.

For the continuous production of TQ-loaded nanodispersions, TQ at three different concentrations of 1.0, 2.5 and 5.0 mg/mL was first dissolved in the already prepared stock ethanol solutions of citrem and SPC, and then the same microfluidic method was applied as described above. The final EtOH concentration and lipid (citrem/SPC mixture) content in the continuously produced nano-self-assemblies are presented in Table 1. It is worth noting that they were produced at a constant citrem/SPC weight ratio of 2:3, and their mean nanoparticle sizes and size distributions were compared with those prepared using the low-energy emulsification batch method described above.

At the employed constant FRR of 20, the two side aqueous streams were mixed with the organic stream [concentrated ethanolic stock solution containing binary citrem/SPC mixture as described above] at a volume ratio of 20:1. This was associated with a significant dilution effect, leading to the formation of TQ-free and TQ-loaded nanodispersions (Table 1) at ethanol and lipid (citrem plus SPC) concentrations of 2.86 and 1.9 wt%, respectively.

In this study, all TQ-free and TQ-loaded nanodispersions were stored at room temperature prior to characterization.

### 2.3. Low-Energy Batch Production of Citrem/SPC Nano-Self-Assemblies

For preparing TQ-free nanodispersions, the recently reported low-energy emulsification method [30] was slightly modified. Briefly, citrem and SPC at appropriate amounts were weighed out and dissolved in EtOH to obtain clear stock solutions of 40 wt% lipid (a binary citrem/SPC mixture prepared at citrem/SPC weight ratio of 2:3) and 60 wt% ethanol. These ethanol solutions were then gently vortexed in excess of 0.01 M phosphate buffer (pH 7.4) for 5 min to produce homogeneous stable milky solutions. The six samples (B1–B6) were prepared at constant 2:3 citrem/SPC weight ratio and their compositions are given in Table 2.

To prepare TQ-loaded nanodispersions, TQ at different concentrations ranging from 1 to 10 mg/mL was first dissolved in ethanol solutions containing binary citrem/SPC mixtures, and then the samples were prepared as described above. The resulting nanodispersions contained a constant EtOH concentration of 1.09 wt% and total lipid (citrem/SPC mixture) content of 8 wt% (samples B1–B4, Table 2). For gaining further insight into the effect of ethanol concentration on the structural features of the produced nanoparticles, additional nanodispersions (samples B5 and B6, Table 2) were prepared at a constant TQ concentration of 2.5 mg/mL and different EtOH contents (in the range of 0–5.45 wt%).

### 2.4. NTA Measurements

Size characterization of the TQ-free nanoparticles produced using the commercial HFF chip and the corresponding nanoparticles prepared by employing the aforementioned batch emulsification method was conducted by using NanoSight NS300 equipped with a 405 nm laser and a microscope (Malvern Panalytical Ltd., Worcestershire, UK). Prior to measurements, the samples were diluted 10^4^ times in filtered PBS pH 7.4 prepared from ultra-pure water (18.2 MΩ cm) to reach a nanoparticle concentration between 10^8^ and 10^9^ nanoparticles/mL. All samples were size measured in triplicate at room temperature and the data represent the obtained average values of these runs. The measurements were based on individually tracked nanoparticles from 5 to 9 videos from different locations in the sample. The recorded videos were analysed using Malvern software (NTA 3.2 Dev Build 3.2.16). PBS pH 7.4 was measured as a control at identical instrumental settings.

### 2.5. Synchrotron Small Angle X-ray Scattering (SAXS)

SAXS investigations were conducted at the Austrian SAXS beamline (Elettra synchrotron facility, Trieste, Italy). An X-ray beam with a wavelength of 1.54 Å (8 keV) was used, with a sample-to-detector distance of 1314 mm, covering a *q*-range (*q* = 4*π sinθ/λ*, where λ is the wavelength and *2θ* is the scattering angle) from 0.18 to 5.00 nm^−1^. Silver behenate (CH_3_-(CH_2_)_20_-COOAg with a *d-*spacing value of 58.38 Å) was used as a standard to calibrate the angular scale of the measured intensity. In these investigations, 1 mm diameter quartz capillaries (sample holders) were used, and the samples were thermostated with a water bath (temperature stability ± 0.1 °C, Unistat CC, Huber, Offenburg, Germany). The SAXS measurements were performed at 25 or 37 °C with an exposure time of 20 s per frame with 3 s delay between 5 frames. The 2D SAXS patterns were acquired using a Pilatus3 1 M detector (Dectris Ltd., Baden, Switzerland; active area 169 × 179 mm^2^ with a pixel size of 172 × 172 μm^2^), integrated into one-dimensional (1-D) scattering function *I*(*q*) by using SAXSdog [56], the data reduction pipeline available at the Austrian SAXS beamline, and then analyzed with IGOR pro (Wavemetrics, Inc., Lake Oswego, OR, USA). After the raw data had been corrected for detector efficiency and background scattering, the averaged scattering patterns of the five acquired frames were plotted [intensity, *I*(*q*), as a function of *q*], and all Bragg peaks were fitted by Lorentzian distributions. The lattice parameters, *ɑ*, of the detected liquid crystalline nanostructures were obtained by calculating the characteristic distance (*d* = 2π/*q*) for every detected Bragg peak, and the corresponding lattice parameters of lamellar (L_α_) and non-lamellar liquid crystalline phases. The detected non-lamellar liquid crystalline phases are an inverse bicontinuous cubic (Q_2_) phase of symmetry *Pn3m*, and an inverse hexagonal (H_2_) phase.

## 3. Results and Discussion

### 3.1. Size Characteristics of TQ-Free and TQ-Loaded Citrem/SPC Nano Self-Assemblies

For gaining insight into the size characteristics of TQ-free and TQ-loaded nano-self-assemblies produced using microfluidics at citrem/SPC weight ratio of 2:3, a set of NTA measurements were performed on nanodispersions produced at a constant FRR of 20 and two different TFRs of 50 and 100 µL/min and constant concentrations of ethanol and lipid (citrem plus SPC). These concentrations were 2.86 and 1.9 wt%, respectively. In absence of TQ (samples A1 and A2), the mean (±SD) nanoparticle sizes (diameters) of the two produced nanodispersions at TFR of 100 and 50 µL/min were ranging from 115.5 ± 42 to 119.7 ± 42 (Table 1). These mean sizes and modes were comparable with those previously reported for ethanol-free citrem/SPC nanodispersions prepared at same citrem/SPC lipid composition by using two different batch methods: a low-energy emulsification method (vortexing for few minutes) and a high-energy emulsification method based on ultrasonication [30,33]. In addition to a possible effect of ethanol on size characteristics, it is important to take into account that citrem is a commercial lipid and a batch-to-batch variability in its composition could contribute to slight alterations in the size characteristics and structural features of this family of citrem/SPC nanoparticles, as discussed in a recent report [33]. On loading TQ at concentrations in the range of 1–5 mg/mL (samples A3–A8, Table 1), the mean (±SD) nanoparticle sizes were ranging from 124.3 ± 38 to 164 ± 54. As presented in Table 1, an increase in the mean nanoparticle sizes and modes was detected on loading TQ to citrem/SPC nanodispersions. This is consistent with previous investigations on the effect of TQ encapsulation on the size characteristics of non-lamellar liquid crystalline nanoparticles based on binary glycerol monooleate/vitamin E mixtures [16], and polymeric nanocarriers [54]. In TQ concentration-dependent manner, the alterations in size characteristics of the former nanoparticles was attributed to TQ-meditated structural transitions [16]. An increase in the mean nanoparticle sizes was also reported on loading other poorly water-soluble drugs to citrem/SPC nanoparticles and other non-lamellar liquid crystalline nano-self-assemblies [28,57].

In addition to the effect of drug loading on mean and mode nanoparticle sizes, it was of interest to shed light on the effect of the batch emulsification method on selected nanodispersions. The NTA findings presented in Table 1 indicated that the mean nanoparticles sizes and modes of TQ-free and TQ-loaded citrem/SPC nano-self-assemblies prepared by microfluidics (samples A1 and A5, respectively, Table 1) were comparable with those of the counterpart nano-self-assemblies prepared by using the batch method (samples A9 and A10, respectively, Table 1). For instance, the mean (±SD) nanoparticle sizes of TQ-free and TQ-loaded nano-self-assemblies (at TQ concentration of 2.5 mg/mL) produced by microfluidics were 115.5 ± 42 nm (sample A1) and 141.2 ± 49 nm (sample A5). The low-energy emulsification method led to the formation of corresponding TQ-free and TQ-loaded nano-self-assemblies (at same TQ concentration of 2.5 mg/mL) with mean (±SD) sizes of 116.8 ± 45 and 141.2 ± 44 nm for sample A9 and A10, respectively (Table 1).

Despite the attractiveness of the used microfluidic device for continuous production of these nanoparticles at the given experimental conditions and a constant FRR of 20, the findings revealed a lack of efficiency in controlling the mean nanoparticle sizes. A similar lack of efficiency was also reported for Pluronic F127-stabilized hexosomes based on docosahexaenoic acid monoglyceride (MAG-DHA) that were continuously produced using polyimide HFF microfluidic device [45], and for a series of lipid nanoparticles loaded with a small-interfering RNA (siRNA) that were produced using HFF device made of fused silica glass [58]. Clearly, further investigations are needed to introduce more efficient microfluidic platforms for the continuous production of citrem/SPC nanoparticles with controllable sizes and size distributions.

**Figure 2:**
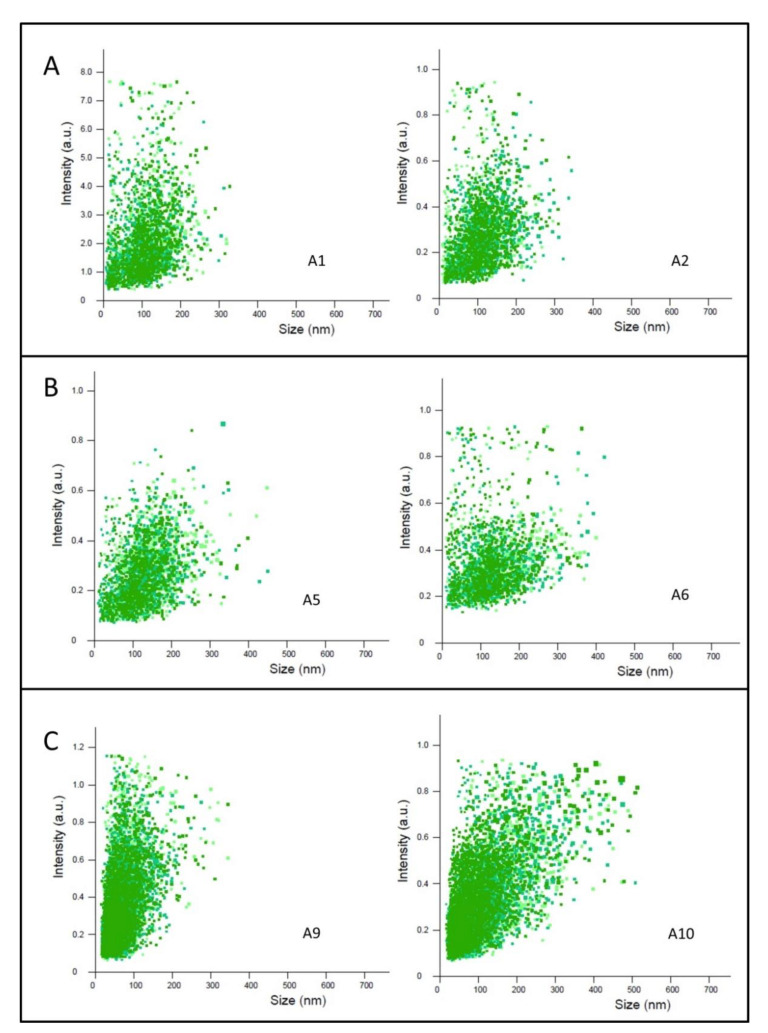
Effects of TQ loading and TFR on nanoparticle size distribution of selected continuously produced TQ-free and TQ-loaded nanodispersions and two nanodispersions prepared using a batch method. 2D plots of relative light-scattering intensities of TQ-free and TQ-loaded citrem/SPC nanoparticles versus nanoparticle sizes are presented. In microfluidic synthesis, all nanodispersions (samples A1, A2, A5, and A6) were prepared at a constant FRR of 20 and two TFRs of 100 and 50 min, respectively. 2D plots of TQ-free (samples A1 and A2) and TQ-loaded (samples A5 and A6) are presented, respectively, in panels (**A**,**B**); whereas panel (**C**) shows the 2D plots of corresponding TQ-free and TQ-loaded nanodispersions (samples A9 and A10) prepared using the low energy-emulsification batch method. All nanodispersions were prepared at citrem/SPC weight ratio of 2:3 and constant EtOH and lipid (SPC plus citrem) concentrations of 2.86 and 1.9 wt%, respectively. TQ-loaded samples were prepared at a constant TQ concentration of 2.5 mg/mL.

In this perspective, to gain further insight on the size characteristics of the continuously produced TQ-free and TQ-loaded nanoparticles, the respective nanoparticle size distributions were investigated by following the NTA 2D plots of relative light scattering intensity of nanoparticles versus their sizes (Figure 2). Here, the effect of varying TFR from 100 to 50 µL/min on size distributions of TQ-free (samples A1 and A2, Figure 2A) and TQ-loaded samples prepared at TQ concentration of 2.5 mg/mL (samples A5 and A6, Figure 2B), respectively, was investigated. As compared to nanoparticle size distributions of corresponding nanodispersions prepared using the low energy-emulsification batch emulsification method (samples A9 and A10, Figure 2C), the 2D plots of the corresponding nanoparticles produced using microfluidics (samples A1 and A2, Figure 2A) indicate roughly comparable nanoparticle size distributions in TQ-free nanodispersions. However, the batch emulsification method led to a slight increase in nanoparticle subpopulations larger than 300 nm (see the 2D plot for sample A10, Figure 2B) as compared to that produced using the microfluidic device (see the 2D plot for sample A6, Figure 2B). Regarding microfluidic synthesis and consistent with previous reports [40,45], the obtained 2D plots presented in Figure 2 indicated no significant changes in the nanoparticle size distributions of TQ-free and TQ-loaded nanodispersions on varying TFR from 100 to 50 µL/min. However, we observed a slight decrease in the mean (±SD) nanoparticle sizes of TQ-free and TQ-loaded nano-self-assemblies on increasing TFR from 50 to 100 µL/min (Table 1). This is consistent with previous findings [40,45,59,60,61], and most likely attributed to presence of a relatively higher shear stress at TFR of 100 µL/min, leading to self-assembly of both lipids in the presence of relatively higher concentration of ethanol at the intersection of the center and side microfluidic channels. This is associated with a provision of relatively shorter time for ethanol to diffuse out and mix with excess buffer on increasing TFR from 50 to 100 µL/min [40,45,59]. It is worth noting that the aforementioned NTA findings were obtained at a constant FRR of 20. As varying FRR may affect the mean nanoparticle sizes and size distributions of citrem/SPC nanoparticles, the effect of varying FRR on the size characteristics of these nano-self-assemblies warrants further investigation.

Regarding the colloidal stability, all TQ-free and TQ-loaded SPC/citrem nanodispersions prepared in this study (Table 1 and Table 2) by employing the batch (low-energy (vortexing) emulsification procedure) and continuous (microfluidics) methods were visually inspected. There were colloidally stable at 25 °C for at least one month of post-preparation. Consistent with previous reports on citrem-stabilized lamellar and non-lamellar nanodispersions [30,31,62,63], the nanoparticles are stabilized in excess buffer through the electrostatic stabilization mechanism: occurrence of electrostatic repulsions owing to the adsorption of the negatively charged citrem molecules onto the outer surfaces of the dispersed colloidal nanoobjects.

### 3.2. Effects of Loading Drug and EtOH Concentration on Citrem/SPC Nano Self-Assemblies Produced by the Batch Emulsification Method

**Figure 3:**
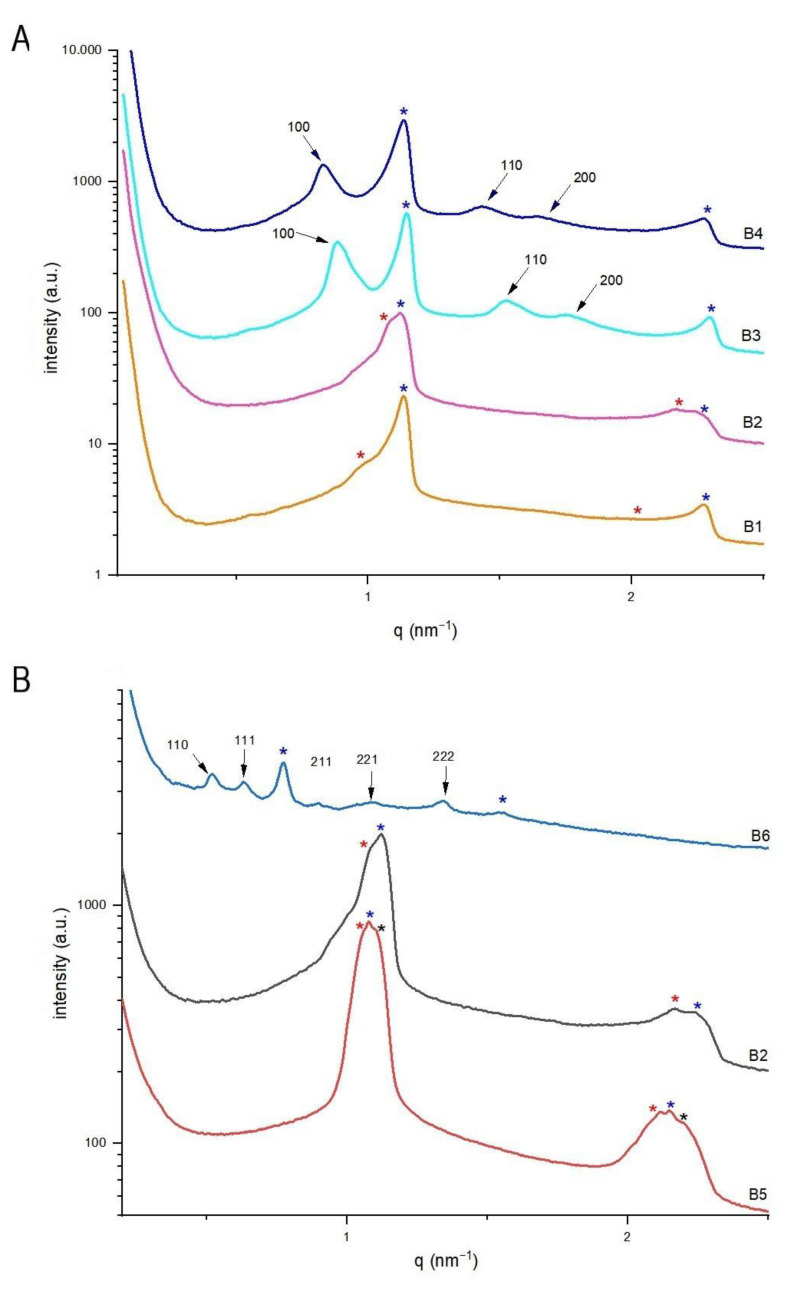
Effects of TQ and ethanol concentrations at 25 °C on the structural features of citrem/SPC nanodispersions prepared using the low energy-emulsification batch method. (**A**) SAXS patterns of samples prepared at TQ concentrations in the range of 1–10 mg/mL. The nanodispersions (B1–B4) were prepared at citrem/SPC weight ratio of 2:3 and constant EtOH and lipid (SPC plus citrem) concentrations of 1.09 and 8.0 wt%, respectively. (**B**) SAXS patterns of 2:3 citrem/SPC nanodispersions prepared at a constant TQ concentration of 2.5 mg/mL and contained different concentrations of ethanol (in the range of 0–5.45 wt%, Table 2). The detected Bragg peaks and corresponding Miller indices for the inverse bicontinuous cubic *Pn3m* (**A**) and inverse hexagonal (H_2_) phases (**B**) are presented and marked with arrows. In panels A and B, the detected two characteristic peaks for the two coexisting L_α(1)_ and L_α(2)_ phases are marked with red and blue asterisks, respectively. The two characteristic peaks of the coexisting third lamellar phase (L_α(3)_ phase) detected in sample B5 is marked with black asterisks.

The effects of TQ loading and ethanol concentrations on the structural features of citrem/SPC nanoparticles were investigated by synchrotron SAXS at 25 °C. Here, we considered the reported sensitivity of citrem/SPC nano-self-assemblies to variations in their lipid composition and loaded drug concentration [28,30,33]. At a constant citrem/SPC of 2:3, the investigated TQ-free and TQ-loaded nanodispersions were prepared by employing the low energy batch emulsification method, and contained lipid (citrem and SPC) and EtOH concentrations of 8.0 and 1.09 wt%, respectively. In absence of TQ (sample B1), it was evident from the SAXS pattern (Figure 3A) that the binary citrem/SPC mixture tends to form multilamellar vesicles (MLVs) in excess buffer as a biphasic feature of two coexisting lamellar (L_α_) liquid crystalline phases was detected. The two characteristic peaks of the first L_α_ phase (L_α(1)_) with *d*-spacing of about 5.55 nm were detected at *q* values of about 1.13 and 2.26 nm^−1^ (marked with red asterisks, Figure 3A). This is consistent with our recent findings on the formation of MLVs with L_α_ phase having a *d*-spacing of 5.71 nm at same lipid composition but in absence of EtOH [30]. There is also an indication on the presence of coexisting traces of a second L_α_ phase (L_α(2)_ with *d*-spacing of 7.22 nm) as identified by the detection of very weak peaks at *q* values of about 0.84 and 1.68 nm^−1^ (marked with blue asterisks, Figure 3A). The formation of the latter swollen L_α(2)_ phase may be attributed to ethanol uptake into the nanoparticles’ interiors. At TQ concentration of 2.5 mg/mL (sample B2), MLVs were still formed and the L_α(1)_/L_α(2)_ biphasic feature was still detected. However, TQ loading was associated with a significant decrease in the *d*-spacing of the L_α(2)_ phase (a decrease from 7.22 to 5.72 nm on loading TQ concentration (Table 2)). Here, we do not exclude that this decrease may be attributed to penetration of TQ into the hydrophobic domains of the L_α(2)_ phase, and a possible release of ethanol molecules. Further increase in TQ concentration to 7.5 (sample B3) and 10 mg/mL (sample B4) was associated with the disappearance of L_α(2)_ phase and formation of coexisting hexosomes. For hexosomes, the identification of the internal inverse hexagonal (H_2_) phase was based on detection of its first three characteristic of Bragg peaks ((100), (110), and (200)), with a spacing ratio of 1:√3:√4, respectively (light and dark blue SAXS patterns, Figure 3A).

In all nanodispersions, it is interesting that the *d*-spacing of the coexisting L_α(1)_ phase remained almost same in the investigated TQ concentration range (0–10 mg/mL), indicating most likely preferential localization of TQ in the swollen L_α(2)_ phase (a possible enhancement of TQ uptake in this ethanol-rich phase). The observed TQ-mediated phase transition from a biphasic L_α(1)_/L_α(2)_ feature to a biphasic feature of L_α(1)_ phase in coexistence with H_2_ phase is most likely attributed to the tendency of TQ to be localized in the hydrophobic domains of the nanoparticles’ self-assembled interiors. However, it was not expected to observe an increase in the lattice parameter, *a*, of the coexisting H_2_ phase from 8.13 to 8.68 nm on increasing TQ concentration from 7.5 to 10.0 mg/mL (Table 2). On loading poorly water-soluble drugs, including TQ, to lamellar and non-lamellar liquid crystalline nano-self-assemblies, it is expected their accommodation into the hydrophobic domains of the nanoparticles’ self-assembled interiors to be associated in a concentration dependent manner with structural changes (a decrease in their lattice parameters), and induction of structural transitions to phases with more negative spontaneous curvatures [16,28,30,33]. This occurs due to simultaneous effects of dehydration of the hydrophilic headgroups of surfactant-like lipids and expansivity of their hydrophobic tails [16,28,30,33,64]. For instance, it was recently reported on a structural transition from a biphasic cubic *Fd3m*/H_2_ feature to a neat cubic *Fd3m* on loading TQ at a concentration of 2.5 mg/mL to TPGS-PEG2000-stabilized nanodispersion based on a binary lipid mixture of monoolein and vitamin E (at a weight ratio of 70:30) [16]. Here, the observed increase in the lattice parameter of coexisting H_2_ phase on increasing TQ concentration is most likely attributed to interactions of TQ with citrem and SPC, leading to an incorporation of certain amount of TQ at the lipid-water interfacial area. Thus, the obtained SAXS results suggest possible accommodation of solubilized TQ in the following two sites (compartments) of SPC/citrem nano-self-assemblies: (i) the hydrophobic domains, and (ii) the interfacial film. A similar lamellar-nonlamellar phase transitions and an increase in lattice parameter of non-lamellar liquid crystalline phases (including the H_2_ phase) was also recently observed on loading the local anesthetic agent bupivacaine to SPC/citrem nanoparticles [28]. Such an increase in lattice parameters was attributed to a partial ionization of bupivacaine, leading to electrostatic interactions among its positively charged molecules and the negatively charged citrem molecules embedded at the lipid-water interfacial area.

To gain insight into the effect of ethanol concentration, the structural features of sample B2 were compared with those prepared at same lipid composition and TQ concentration of 2.5 mg/mL (samples B5 and B6, Table 2). On increasing ethanol concentration from 0 (sample B5) to 1.09 wt% (sample B2), there was a slight change in the *d*-spacing of L_α(1)_ and L_α(2)_ phases but the third additional coexisting lamellar phase (L_α(3)_ phase detected in sample B5) disappeared (Figure 3B). It is interesting that a further increase in ethanol concentration to 5.45 wt% (sample B6) was associated with a significant structural impact. This was evident by an ethanol-mediated transition from biphasic L_α(1)_/L_α(2)_ feature (sample B2) to a relatively highly swollen L_α_ phase with *d*-spacing of 8.14 nm coexisting with cubic *Pn3m* phase with lattice parameter of 17.07 nm (sample B6, Table 2 and Figure 3B). The identification of the latter phase is based on the identification of its first characteristic Bragg peaks ((110), (111), (211), (221), and (222)). Taking into account the tendency of citrem to induce lamellar-nonlamellar structural transition in SPC/citrem nanodispersions [28,30,32,33] in a concentration-dependent manner, it is most likely that increasing ethanol concentration is associated with redistribution of SPC and citrem molecules, leading to SPC-rich nanoparticles with swollen L_α_ phase in coexistence with citrem-rich nanoparticles enveloping an internal cubic *Pn3m* phase.

### 3.3. Structural Characteristics of Continuously Produced Citrem/SPC Nano-Self-Assemblies Using Microfluidics

**Figure 4:**
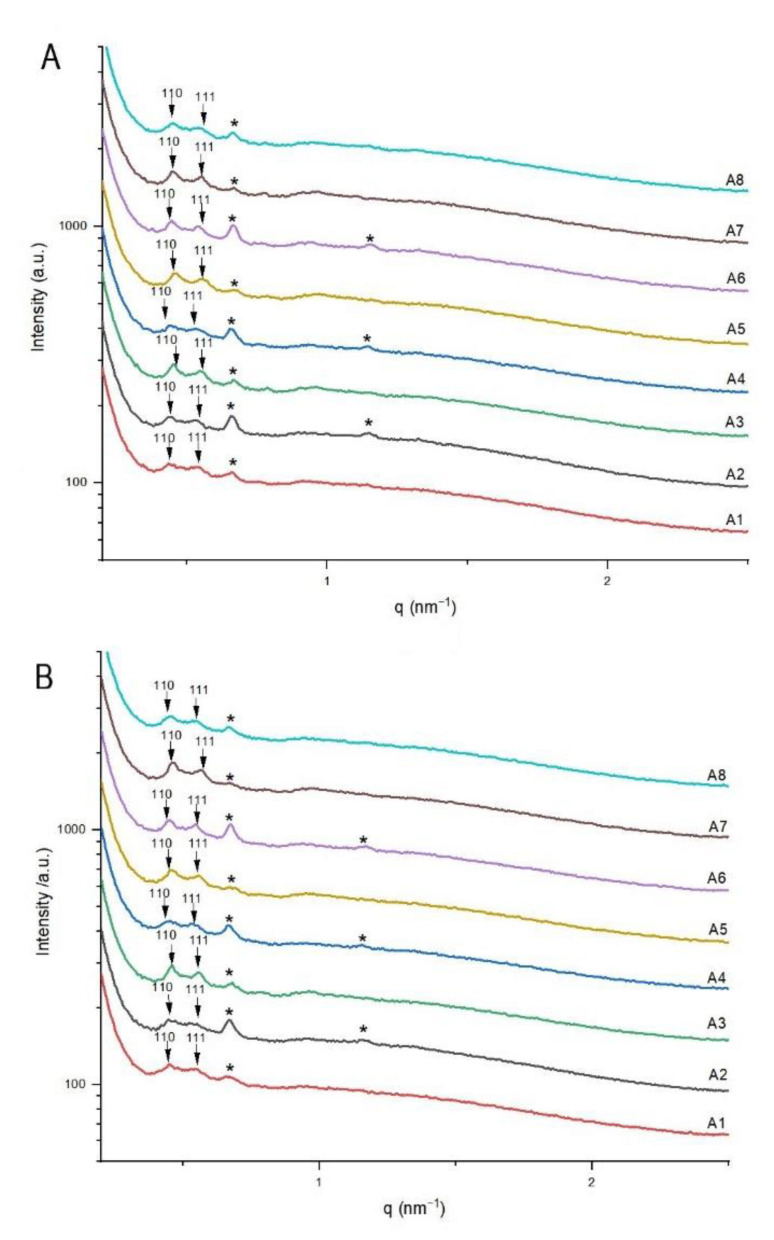
Structural features of continuously produced TQ-free and TQ-loaded citrem/SPC nanodispersions (samples A1–A8, Table 1) using microfluidics. SAXS patterns at 25 (**A**) and 37 °C (**B**). The detected Bragg peaks and corresponding Miller indices for the inverse bicontinuous cubic *Pn3m* are presented and marked with arrows. In panels A and B, the detected two characteristic peaks for the coexisting swollen L_α_ phase are marked with black asterisks. All nanodispersions were produced at citrem/SPC weight ratio of 2:3 and constant ethanol and lipid (SPC plus citrem) concentrations of 2.86 and 1.9 wt%, respectively.

To gain insight into the structural features of SPC/citrem nanodispersions produced using microfluidics, a set of SAXS experiments was conducted at 25 and 37 °C. All nanodispersions were produced at constant ethanol and lipid (citrem plus SPC) concentrations of 2.86 and 1.9 wt%, respectively, by employing a constant FRR of 20 and two different TFRs of 50 and 100 µL/min (Table 1). They were prepared at SPC/citrem weight ratio of 2:3 and contained TQ in a concentration range of 0–5 mg/mL. At 25 (Figure 4A) and 37 °C (Figure 4B), the SAXS patterns of all samples indicated the formation of a biphasic cubic *Pn3m*/swollen L_α_ feature with slight changes in their structural parameters on increasing temperature, varying TFR, and/or increasing TQ concentration. The identification of the former phase was based on the detection of its first two Bragg peaks ((110), (111)). In Figure 4, the detected Bragg peaks for the latter coexisting phase are marked with a black asterisk.

The slight influence of varying temperature, TFR, and TQ concentration on the structural features of the nanodispersions was reflected in the lattice parameters and *d*-spacings of coexisting *Pn3m*/swollen L_α_ phases. For instance, the lattice parameters of the cubic *Pn3m* phase and *d*-spacings of coexisting swollen L_α_ phases at 37 °C were in narrow ranges of 19.18–19.71 and 9.33–9.48 nm, respectively. These results were consistent with the aforementioned findings on the effect of ethanol on citrem/SPC nano-self-assemblies, indicating most likely self-assembly of both lipids at the intersection of the center and side microfluidic channels into SPC- and citrem-rich L_α_ and cubic *Pn3m* phases, respectively. As compared to vesicles based on SPC alone or in combination with small concentrations of citrem that were prepared in absence of ethanol [30,33], the significant increase in *d*-spacing from about 6.28 nm (ethanol-free samples [30,33]) to *>*9.3 nm in all samples (Table 1) is most likely attributed to ethanol uptake by the coexisting SPC-rich L_α_ phase at both TFRs.

## 4. Conclusions

On the basis of limited number of studies on microfluidic synthesis of non-lamellar liquid crystalline drug nanocarriers, we focused on introduction of a simple-by-design commercial microfluidic chip for the continuous production of TQ nanocarriers consisting of a binary lipid mixture of citrem and SPC. Taking into account the reported hemocompatibility and the potential use of these nano-self-assemblies for drug delivery applications [30], we aimed at introducing a simple method for their continuous production. We found that the mean nanoparticles and size distributions are comparable to corresponding nanoparticles prepared using a low-energy emulsification batch method. In addition to nanoparticle size characterization using NTA, the structural features of TQ-free and TQ-loaded nanodispersions were investigated using synchrotron SAXS. Our SAXS results indicated the formation of a biphasic feature of swollen L_α_ phase in coexistence with cubic *Pn3m* phase in all samples produced through microfluidic synthesis. Slight structural changes were associated with varying temperature, TFR, and/or augmenting TQ concentration. In addition, we compared the SAXS findings with those conducted on samples prepared using a batch method, and discussed the effects of ethanol and TQ concentration. Our results reveal the importance of comparing the size characteristics of continuously produced nanoparticles with those prepared using batch methods, and highlight the need for further investigations to introduce efficient microfluidic platforms for the continuous production of cubosomes, hexosomes, and related nano-self-assemblies with controllable sizes and size distributions.

We show that the employed experimental conditions, lipid composition, drug concentration, and ethanol content may modulate the structural features and size characteristics of the produced nanoparticles, and may also dictate the colloidal transformations from lamellar (liposomes) to non-lamellar liquid nano-self-assemblies or vice versa. Thus, it is worth in future investigations to consider detailed characterization studies when proposing novel or improved microfluidic methods for the continuous production of such lipid nano-self-assemblies.

In the development of a next generation of drug nanocarriers and vaccines, further microfluidic design optimization of HFF microfluidic systems and introduction of new 3D microfluidic platforms may contribute to the continuous production of monodispersed nanoparticles having unique internally structural attributes such as cubosomes and hexosomes.

## Figures and Tables

**Table 1:** Nanoparticle size analysis and lattice parameters of identified biphasic features of lamellar (L_α_) phase in coexistence with an inverse bicontinuous cubic *Pn3m* phase. For determination of the structural features of the produced nano-self-assemblies, SAXS experiments were conducted at two different temperatures: 25 and 37 °C; whereas NTA measurements were performed for investigating their size characteristics.

Sample Name ^a^	TQ (mg/mL)	TFR (µL/min)	Space Group	Lattice Parameter (nm ± Error%) 25 °C 37 °C		Size (nm) ^c^ Mean ± SD Mode ± SE	
**A1**	0	100	L_α_ *Pn*3*m*	9.63 20.18 ± 0.03	9.48 19.70 ± 0.05	115.5 ± 42	134.8 ± 3.6
**A2**	0	50	L_α_ *Pn*3*m*	9.51 20.18 ± 0.03	9.39 19.70 ± 0.05	119.7 ± 42	114.4 ± 3.8
**A3**	1	100	L_α_ *Pn*3*m*	9.42 19.62 ± 0.02	9.43 19.36 ± 0.02	124.3 ± 38	126 ± 5.5
**A4**	1	50	L_α_ *Pn*3*m*	9.56 20.18 ± 0.03	9.43 19.70 ± 0.05	139.8 ± 48	136.7 ± 3.2
**A5**	2.5	100	L_α_ *Pn*3*m*	9.52 19.26 ± 0.04	9.36 19.27 ± 0.03	141.2 ± 49	145.5 ± 6.0
**A6**	2.5	50	L_α_ *Pn*3*m*	9.46 19.87 ± 0.02	9.33 19.74 ± 0.02	150.1 ± 61	140.3 ± 2.7
**A7**	5	100	L_α_ *Pn*3*m*	9.46 19.6 ± 0.02	9.44 19.18 ± 0.01	147.7 ± 45	150.5 ± 7.
**A8**	5	50	L_α_ *Pn*3*m*	9.48 19.71 ± 0.04	9.41 19.62 ± 0.06	164 ± 54	144.6 ± 8.1
**A9**	0	Batch	N.I.	N.I. ^b^		116.8 ± 45	102.3 ± 14.1
**A10**	2.5	Batch	N.I.	N.I. ^b^		141.2 ± 44	135.1 ± 3.7

^a^ All nanodispersions were produced at a constant FRR of 20, and constant ethanol and lipid (citrem plus SPC) concentrations of 2.86 and 1.9 wt%, respectively. ^b^ Samples A9 and A10 were not investigated (N.I.). ^c^ Mean and mode nanoparticle sizes are presented with standard deviations and standard errors, respectively.

**Table 2:** Structural features of citrem/SPC nano-self-assemblies produced by employing a low-energy batch emulsification method. For investigating the effects of EtOH and TQ concentrations, synchrotron SAXS experiments were conducted at 25 °C.

Sample Name ^a^	EtOH %	Lipid %	TQ (mg/mL)	Space Group	Lattice Parameter (nm ± Error%)
**B1**	1.09	8.0	0	L_α(1)_ L_α(2)_	5.55 ± 0.06 7.22 ± 2.63
**B2**	1.09	8.0	2.5	L_α(1)_ L_α(2)_	5.61 ± 0.05 5.72 ± 0.24
**B3**	1.09	8.0	7.5	L_α_ H_2_	5.50 ± 0.06 8.13 ± 0.24
**B4**	1.09	8.0	10.0	L_α_ H_2_	5.56 ± 0.02 8.68 ± 0.27
**B5**	0	9.09	2.5	L_α(1)_ L_α(2)_ L_α(3)_	5.93 ± 0.006 5.83 ± 0.06 5.75 ± 0.13
**B6**	5.45	3.64	2.5	L_α_ *Pn*3*m*	8.14 ± 0.02 17.07 ± 0.01

^a^ All nanodispersions were prepared at a constant EtOH and lipid (SPC plus citrem) content of 9.09%.

## Data Availability

Not applicable.
